# Supplementary material for: Phenotypic diversification by enhanced genome restructuring after induction of multiple DNA double-strand breaks
Source: Nat Commun. 2018 May 18;9:1995. doi: 10.1038/s41467-018-04256-y (PMC5959919; doi:10.1038/s41467-018-04256-y)
Supplement: Supplementary file 1 — Supplementary Information [file 41467_2018_4256_MOESM1_ESM.pdf]

Supplementary Information

Phenotypic diversification by enhanced genome restructuring after induction of multiple DNA double strand breaks

Muramoto et al.

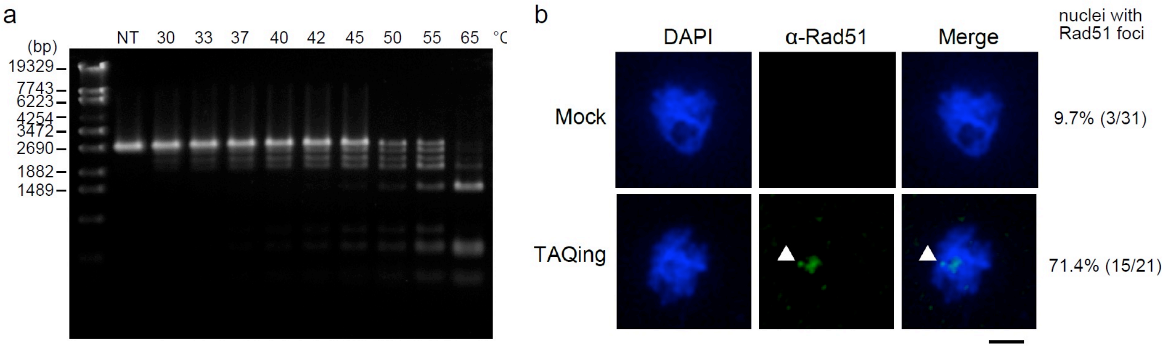

Supplementary Fig. 1. Heat-dependent TaqI activation. (**a**) *In vitro* TaqI-activation assay. *Nde*I-linearized pUC19 plasmid DNA (2686-bp, second lane) was incubated with purified TaqI for 30 min at the indicated temperatures. (**b**) Rad51 foci formation in TAQed cells. TaqI expression was induced in wild-type cells expressing pTaqI (indicated as "TAQing") or vector controls ("Mock"), and foci formation was monitored by immunostaining. Arrowheads indicate Rad51 foci. Foci frequency is shown on the right. Bar, 2 μm.

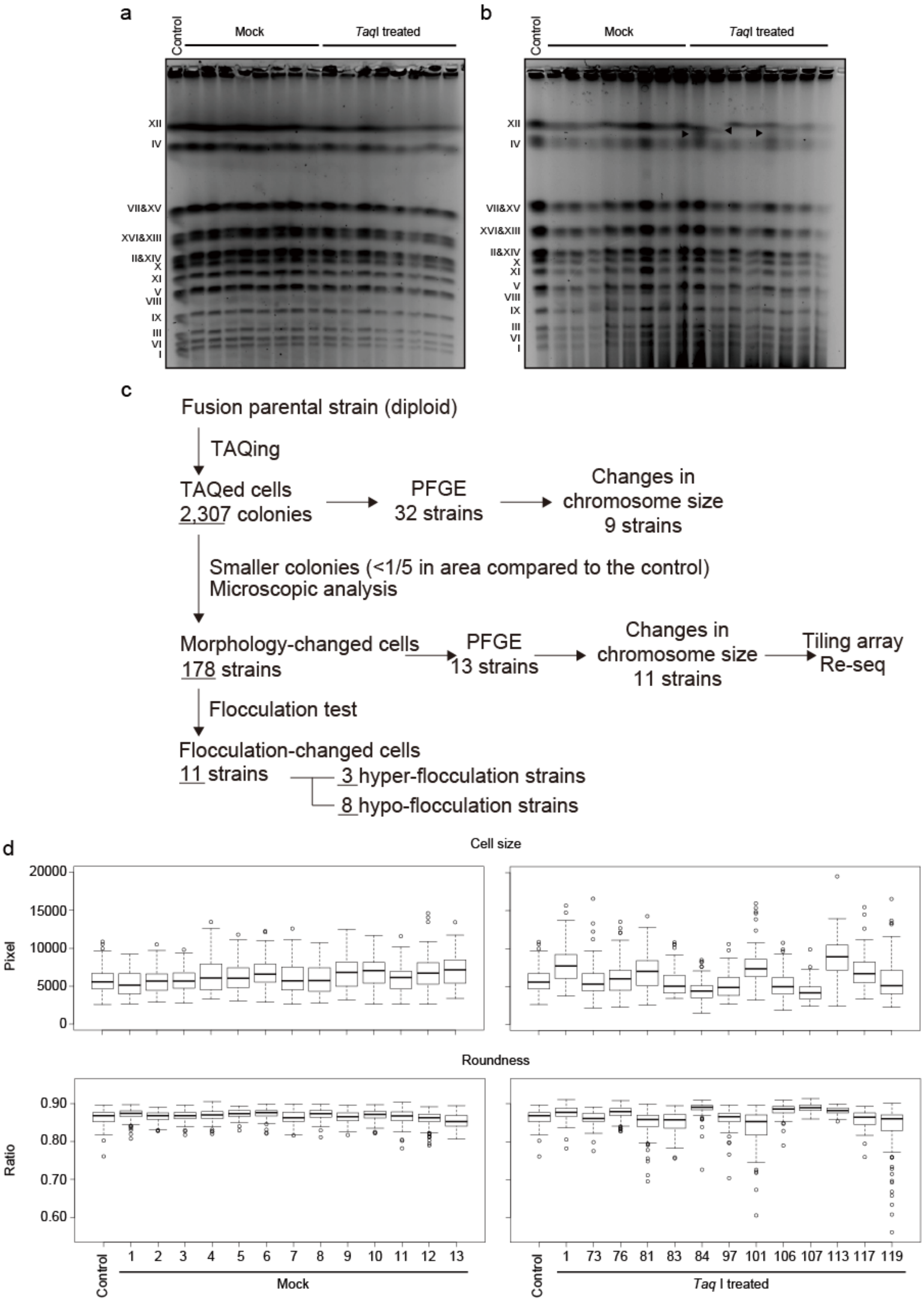

Supplementary Fig. 2. TAQed mutant showing various phenotypic changes. (**a, b**) Differences in chromosome size revealed by PFGE in (**a**) mock-treated or TAQed haploid (YPH499) and (**b**) fused diploid (WT14) strains. Arrowheads indicate chromosozsmal bands exhibiting size changes. (**c**) Workflow of TAQed-mutant selection. (**d**) Variability in cell area (upper) and roundness (lower) after TAQing treatment. *n* = 100, the center line is the median, bounds are the 25th and 75th percentiles and whiskers are ± 1.5 IQR.

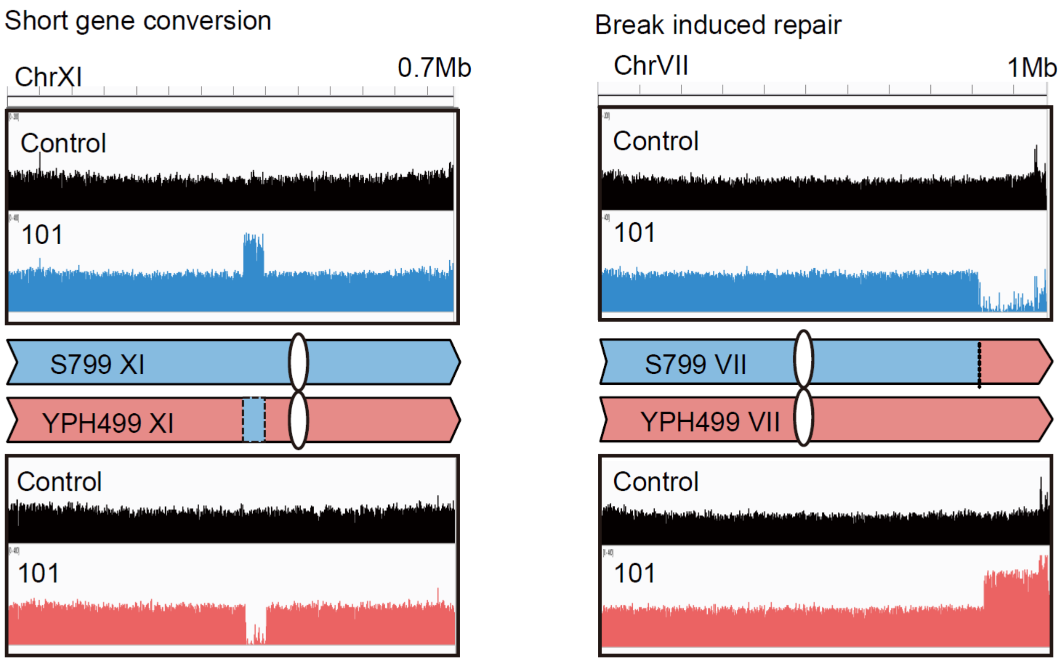

Supplementary Fig. 3. Chromosome structures in TAQed yeast strains. Chromosome-wide mapping data for control (black) and TAQed strains (S799, blue; YPH499, red) exhibiting typical chromosomal rearrangements: SGCs and BIRs.

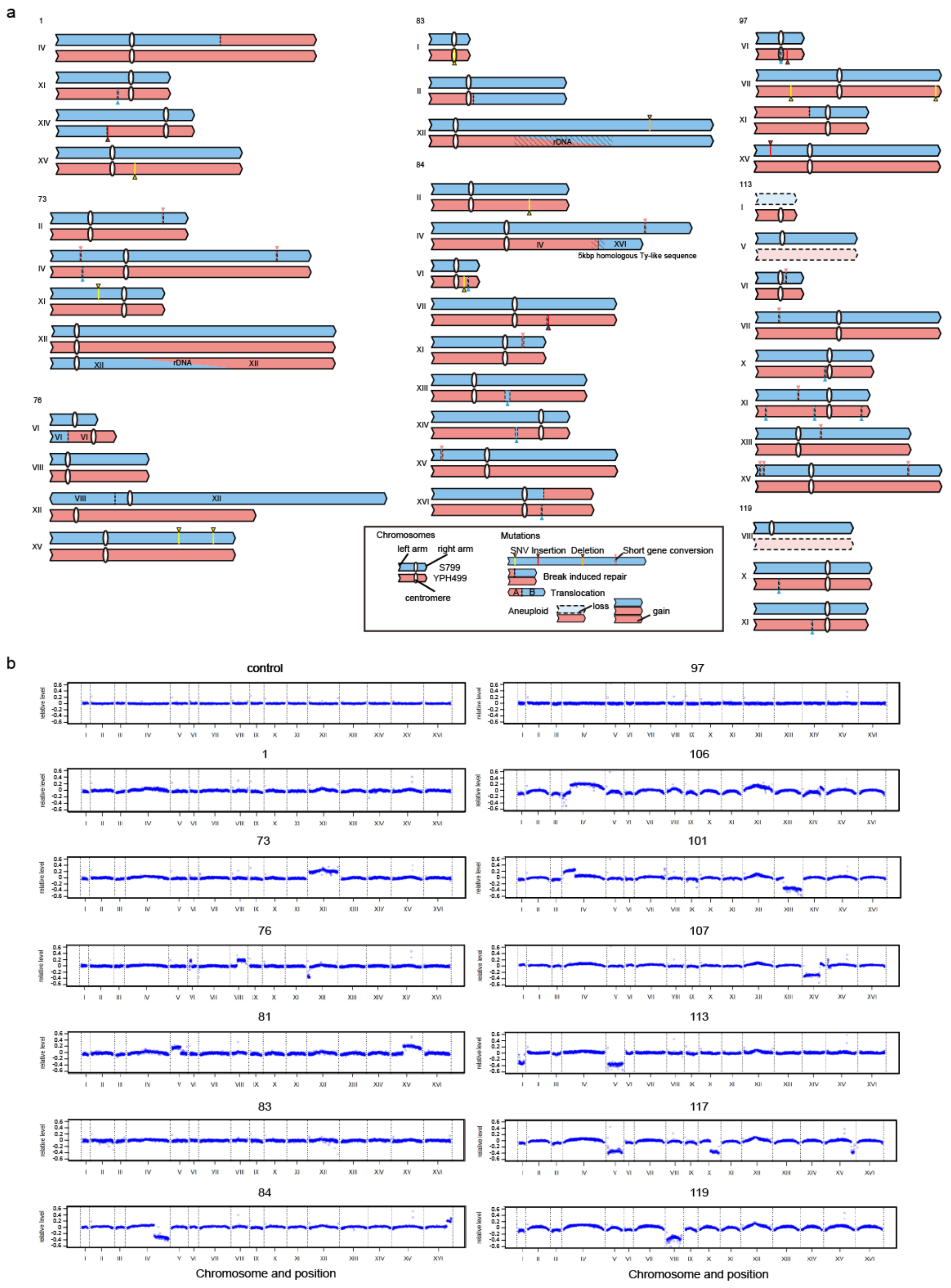

Supplementary Fig. 4. Genomic rearrangement of TAQed yeast strains. (**a**) Schematic diagrams of mutated chromosomes in TAQed strains 1, 73, 76, 83, 84, 97, 113, and 119. Only chromosomes with mutations or aneuploidies are shown. (**b**) aCGH analysis of the TAQed yeast strains used in this study. Genomic DNA from each strain was compared to controls (WT14: cell-fusion strain without TAQing treatment), and $\log_{10}$ DNA copy number ratios were plotted. Data were cropped at a $\log_{10}$ ratio of ~0.6 and averaged across each chromosome using a sliding window of 10 oligonucleotides.

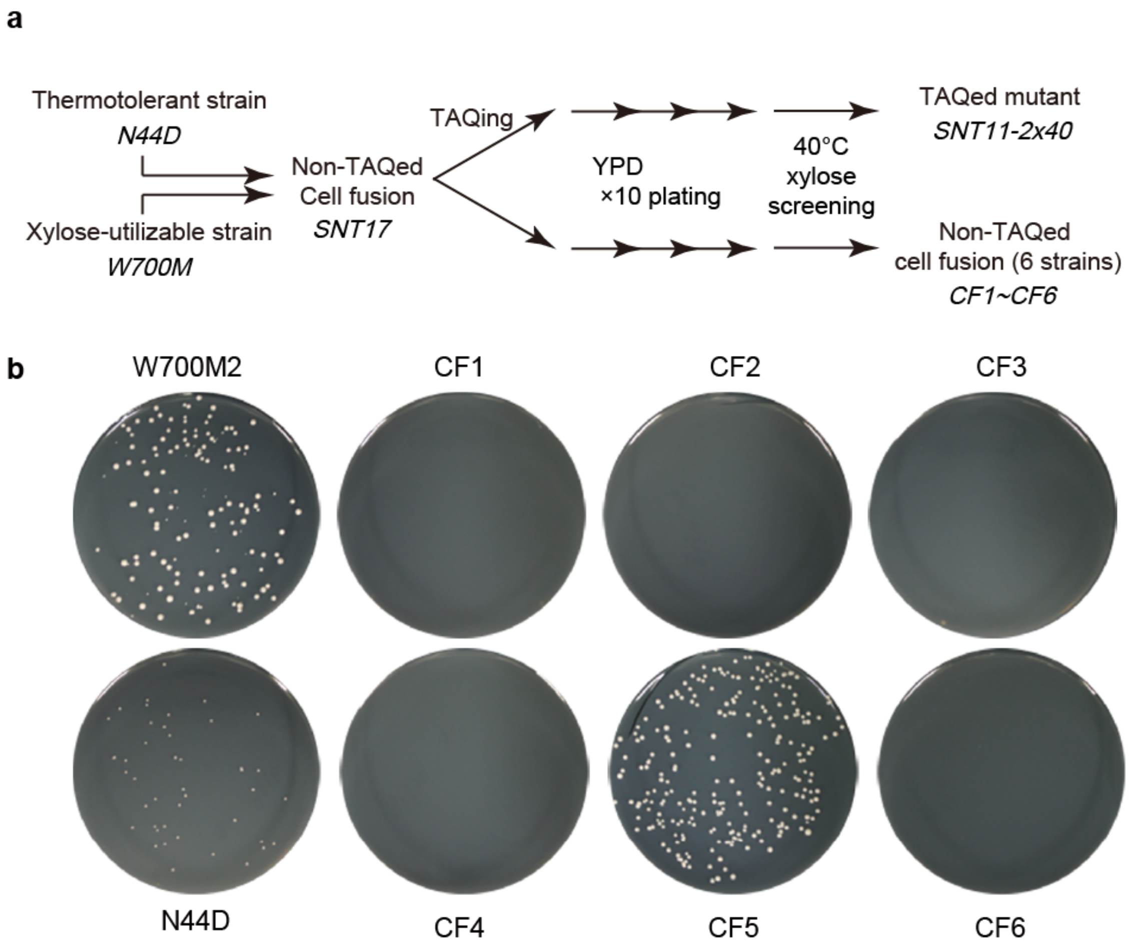

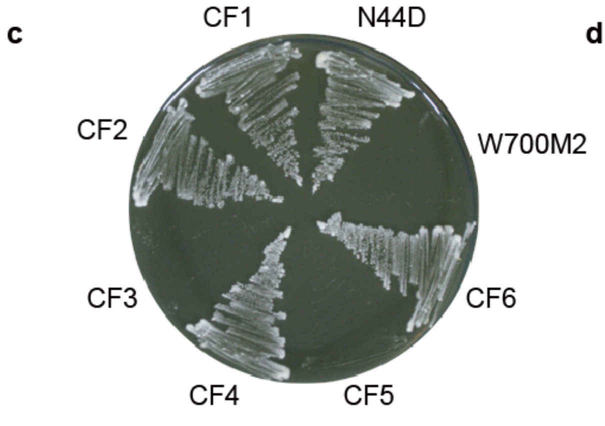

d

strains	xylose, 30 °C	glucose, 40 °C
W700M2	+	-
N44D	±	+
CF1	-	+
CF2	-	+
CF3	-	-
CF4	-	+
CF5	+	-
CF6	-	+

Supplementary Fig. 5. Growth phenotype of cell-fusion mutants. (**a)** Schematic image of xylose-utilizable, thermotolerant TAQed-mutant selection. **(b, c**) Photos show growth phenotype on (**b**) xylose agar plates at 30ºC and (**c**) YPD agar plates at 40ºC for 48 h. Xylose-fermentable strain (W700M2), heat-resistant parental strain (N44D), and non-TAQed cell-fusion strains (CF1–6). (**d**) Summary of growth on agar plates in the presence of xylose or glucose at 30ºC or 40ºC after 10 rounds of plating. Growth phenotypes: extensive (+), moderate (±), and weak (−).

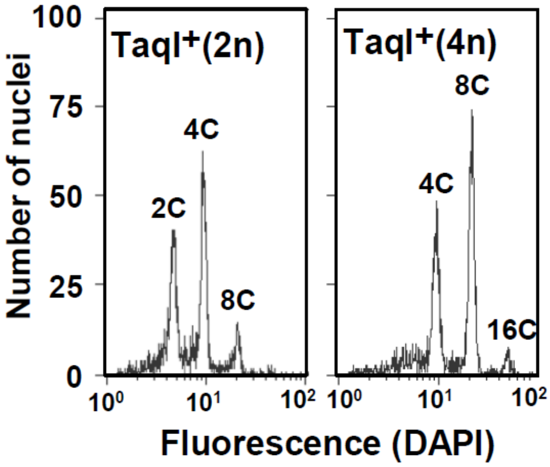

Supplementary Fig. 6. Colchicine-induced tetraploidization was examined in true leaf cells derived from 3-week-old TaqI^{+}(2n) and TaqI^{+}(4n) plants by flow cytometry. Each peak indicates a nuclear phase (2C, 4C, 8C, and 16C).

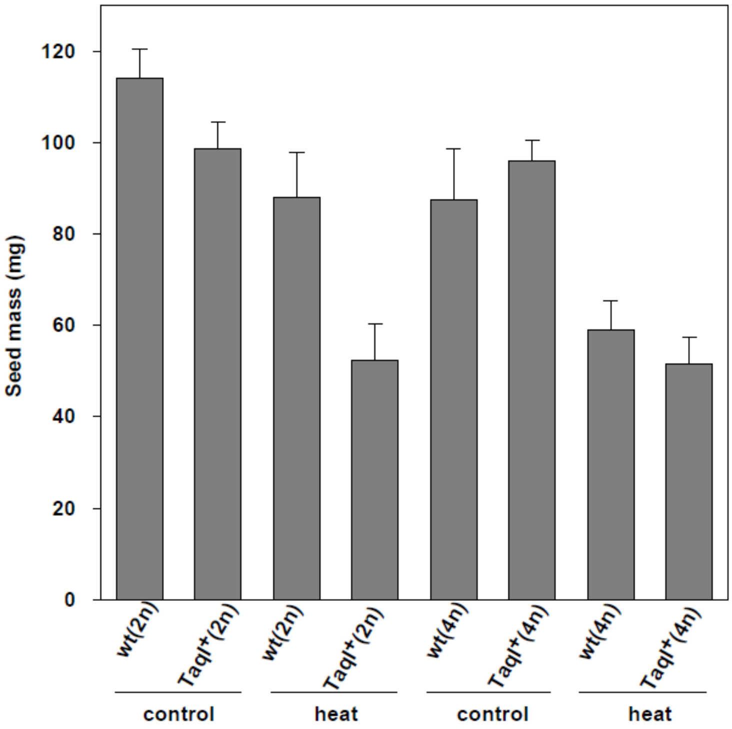

Supplementary Fig. 7. Seed mass of TaqI^{+}(2n) and TaqI^{+}(4n) plants. Data show the yields of seeds from plants grown in soil for 14 weeks along with heat treatment (heated at 37ºC for 24 h in 1-week-old plants) or without heat treatment (control). Data represent the mean ± SEM (*n* = 7–10/strain).

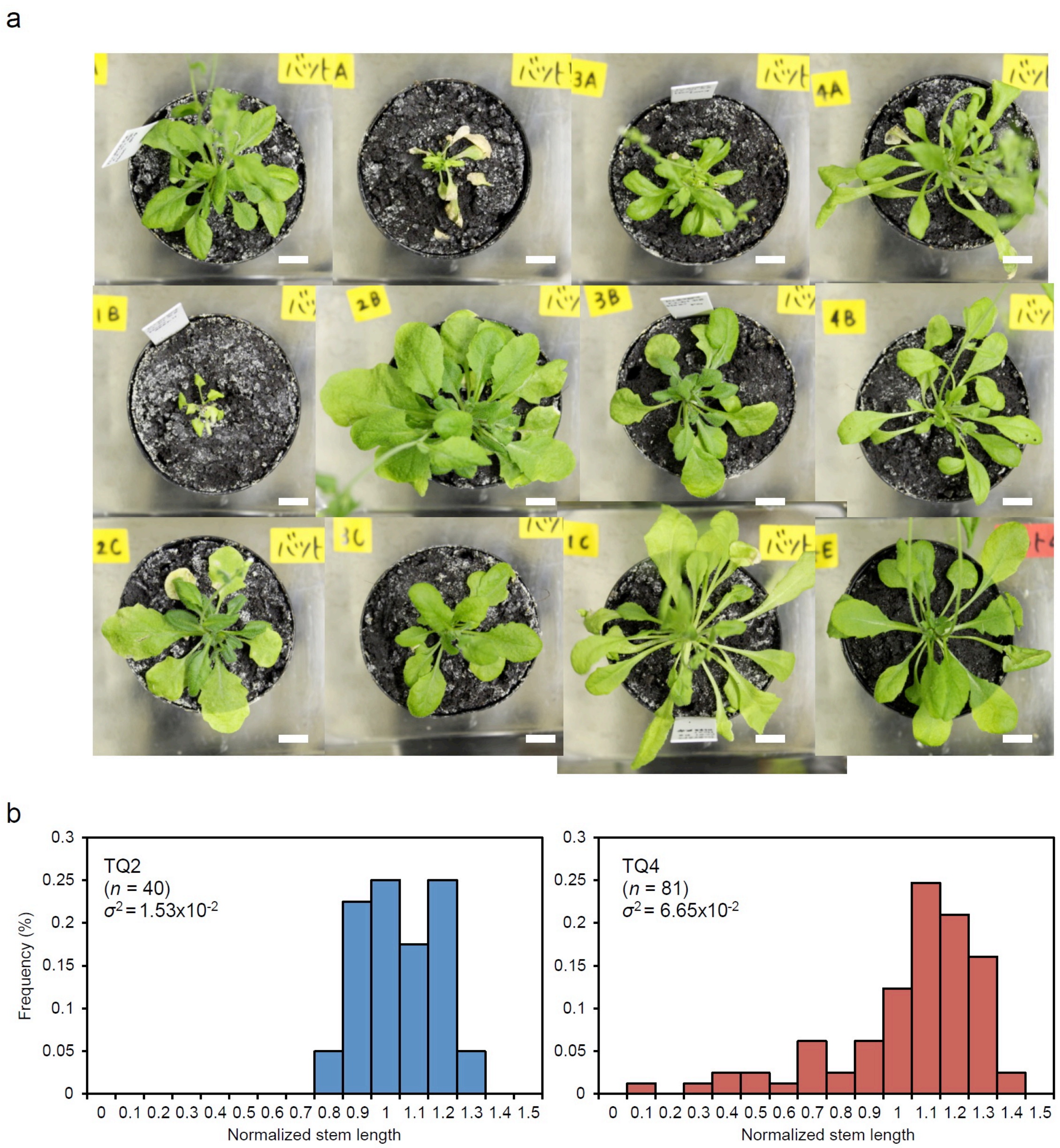

Supplementary Fig. 8. Phenotypic divergence of TQ4 plants. (**a**) Representative TQ4 plant morphology. The upper left image shows wt(4n) plants, and the others show TQ4 mutant plants grown in soil for 49 days. Bars, 1 cm. (**b**) Divergence of stem length in TQ2 and TQ4 plants grown in soil for ~9 weeks. The stem length was measured and normalized to an average of 1.0. σ^2 represents the variance in the normalized stem length. The variance in the TQ4 plants was significantly larger than that in the TQ2 plants $P < 0.01$; F-test).

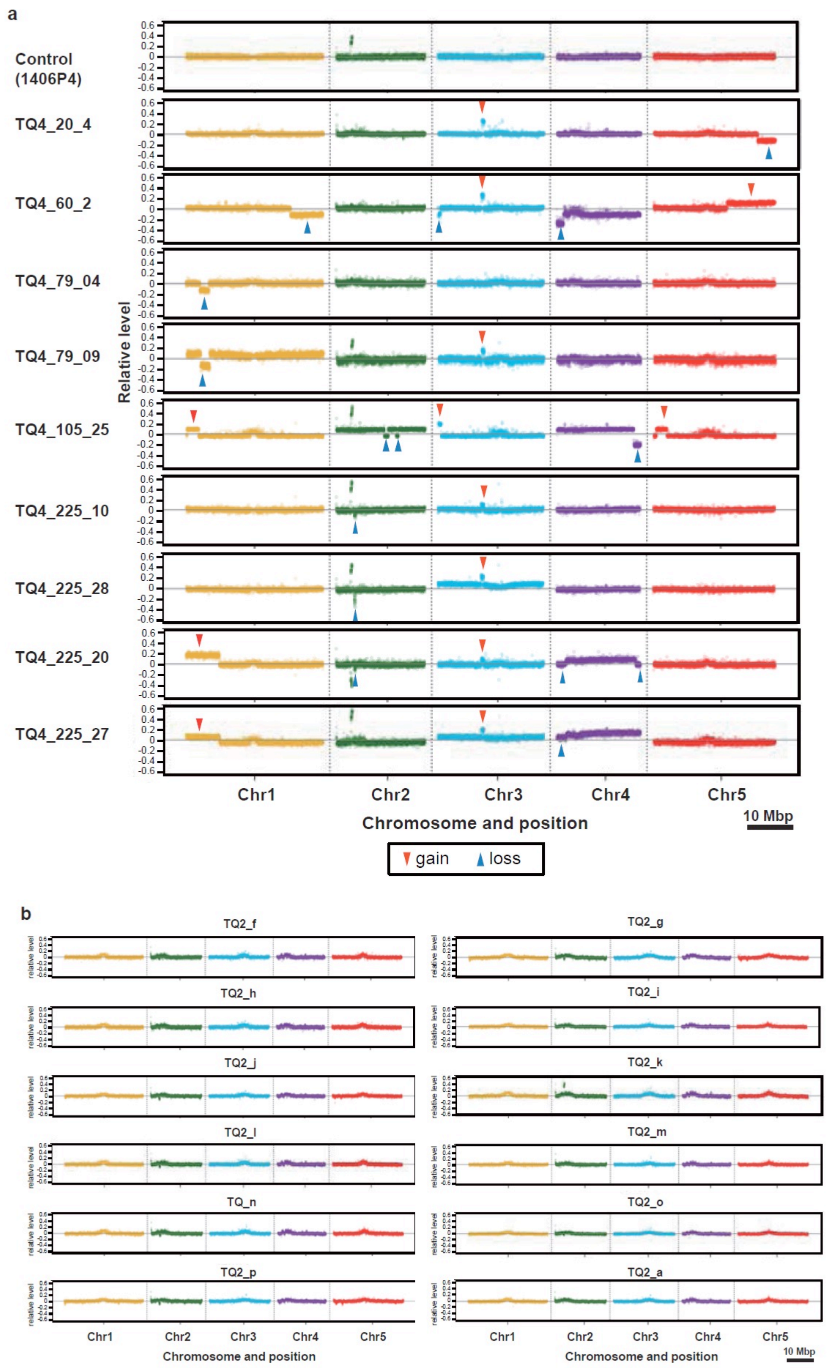

Supplementary Fig. 9. aCGH analysis of (**a**) TQ4 and (**b**) TQ2 plants. Genomic DNA from each strain was compared with that of controls [wt(2n) and wt(4n) for TQ2 and TQ4, respectively], and $\log_{10}$ DNA copy number ratios were plotted. To interpret regions of large CNVs, data were cropped at a $\log_{10}$ ratio of ±0.6 and averaged across each chromosome using a sliding window of 10 oligonucleotides. Red arrowheads: increase in CNVs; blue arrowheads: decrease in CNVs.

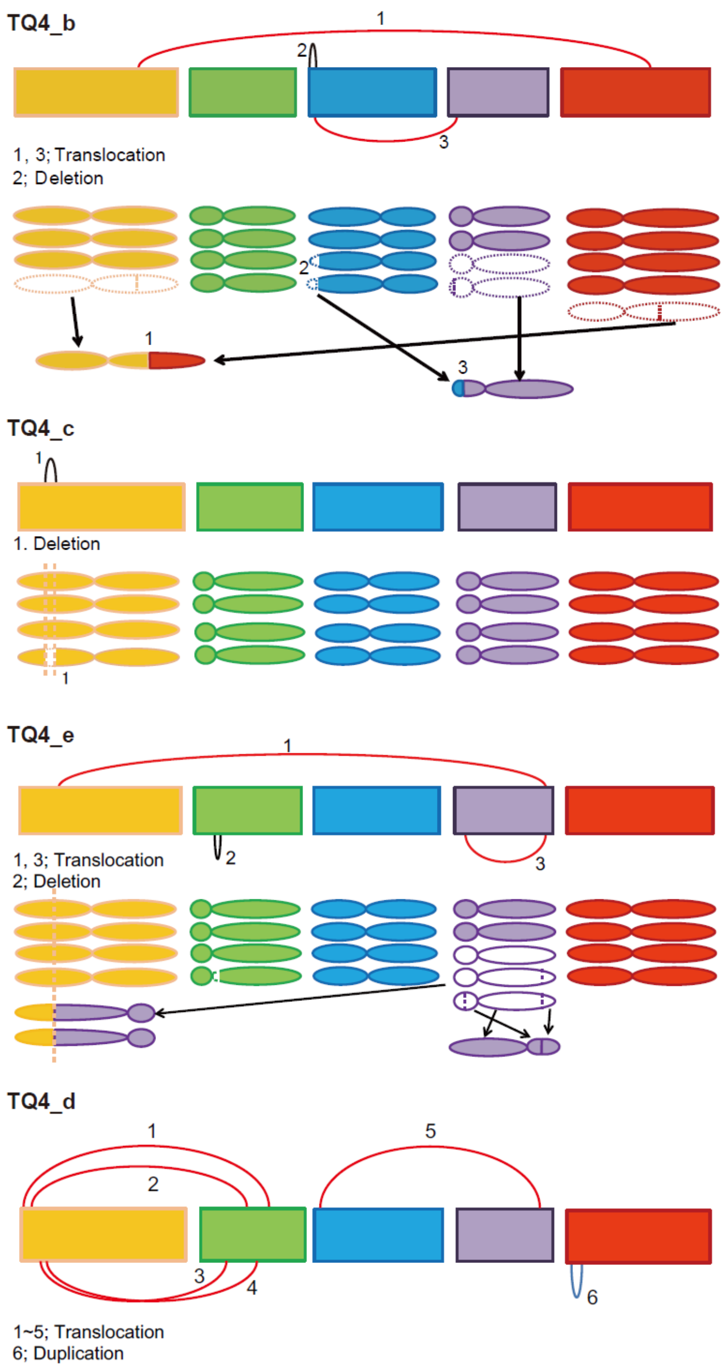

Supplementary Fig. 10. Estimated chromosome structure in each TQ4 plant based on genome-rearrangement analysis.

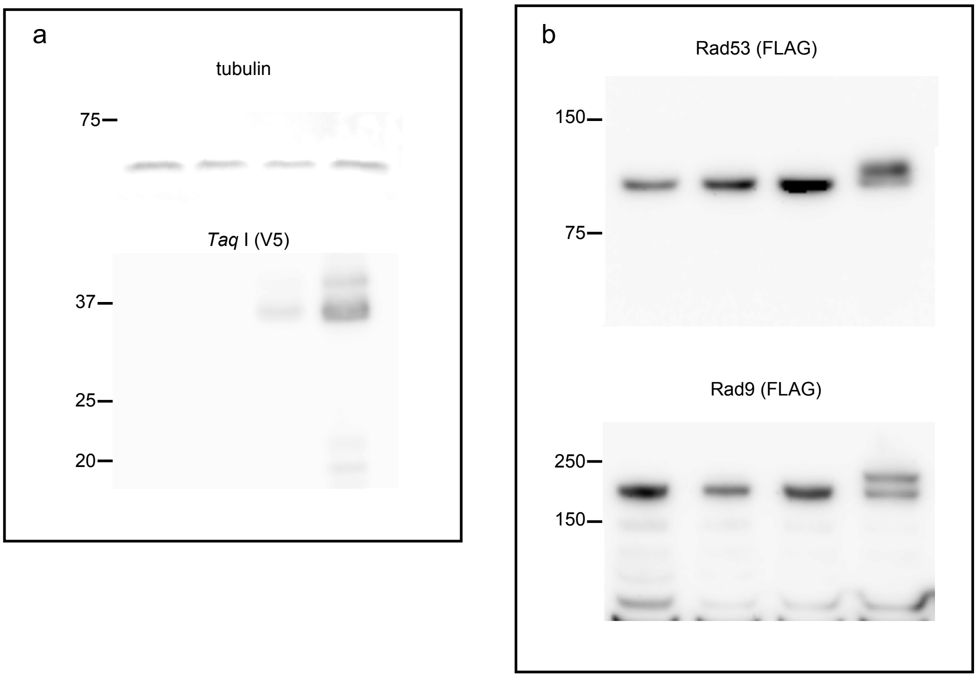

Supplementary Fig. 11. Uncropped blots. **(a)** Uncropped blots from Fig. 1b. **(b)** Uncropped blots from Fig. 1c.

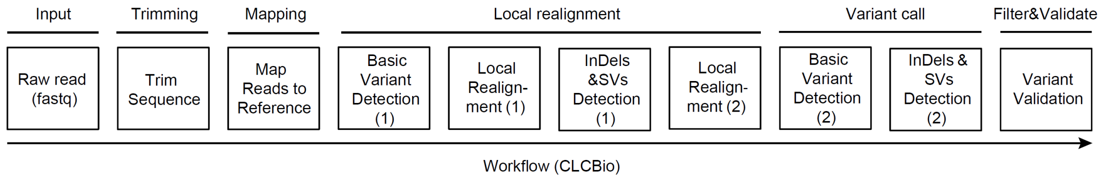

Supplementary Fig. 12. Workflow of variant detection in the *Arabidopsis* genome. Sequenced reads of TAQed plant genomes were mapped using commercial software (CLC Genomics Workbench v8.5; Qiagen).

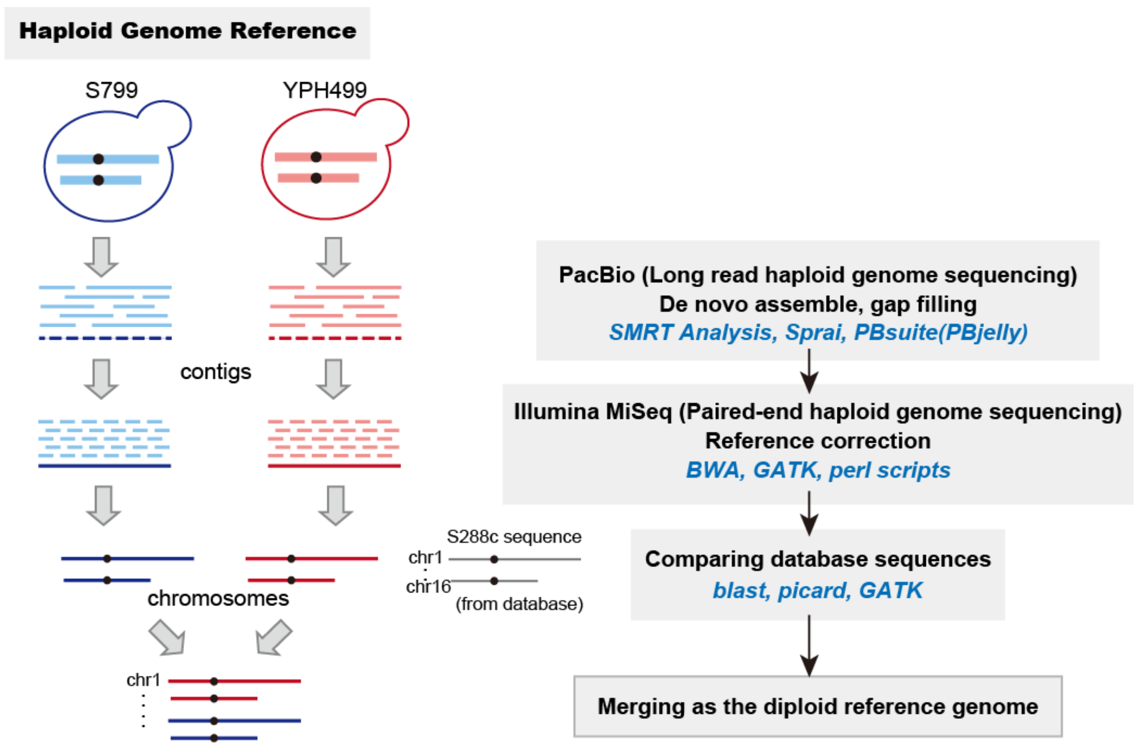

Supplementary Fig. 13. Bioinformatics workflow of yeast parental haploid-genome sequencing. *De novo* assembly was performed using PacBio data and corrected using MiSeq data.

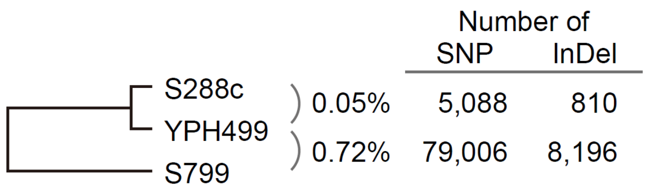

Supplementary Fig. 14. Numbers of SNPs and InDels between parental haploid strains. Genomic differences between S288c and YPH499, and YPH499 and S799 are shown.

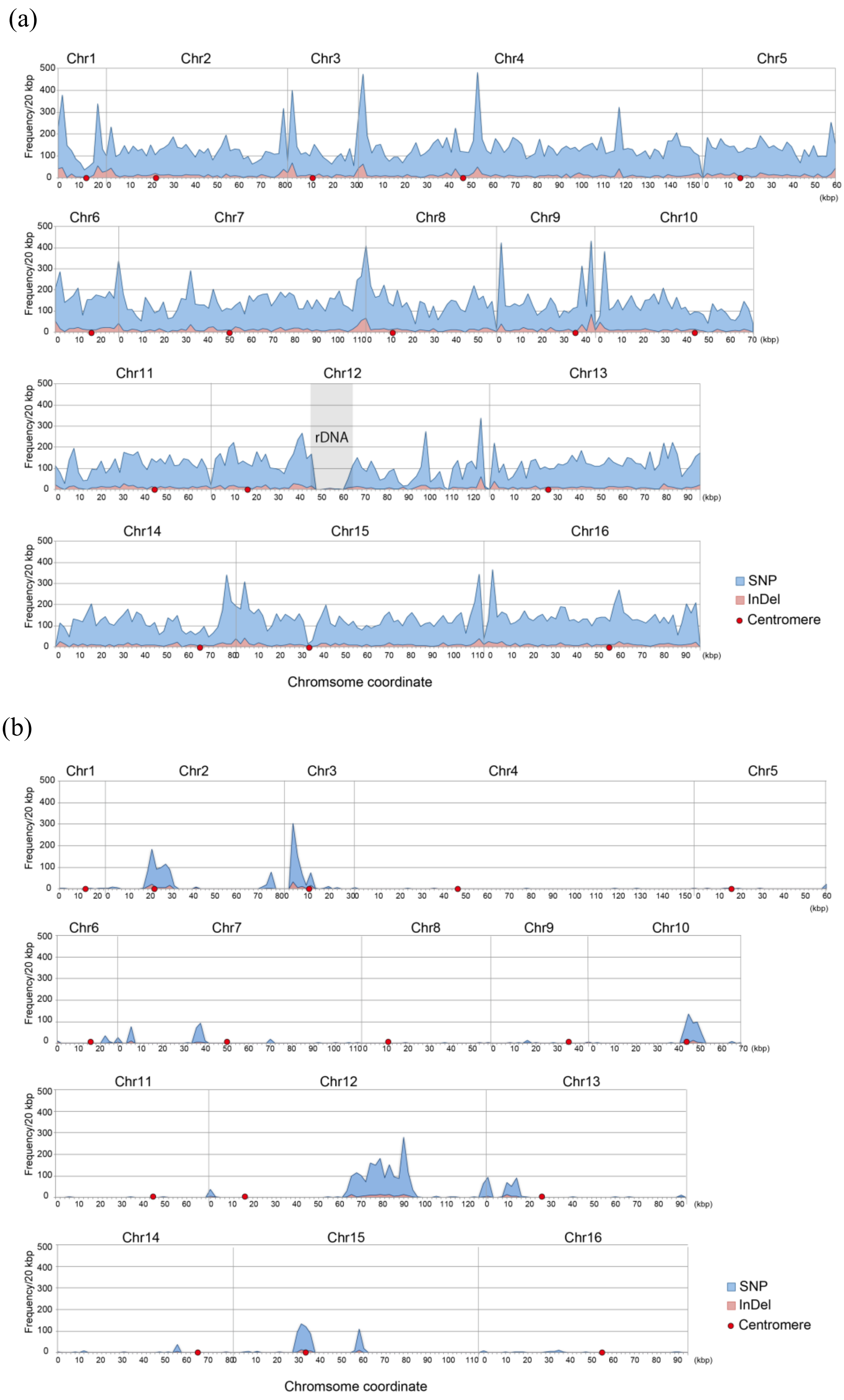

Supplementary Fig. 15. Distribution of SNPs and InDels between strains. SNP and InDel density among the 16 chromosomes between (**a**) S799 and YPH499 (average: 6.5 SNPs/kb and 0.7 InDels/kb) and b) YPH499 and S288c (average: 0.4 SNPs/kb and 0.07 InDels/kb).

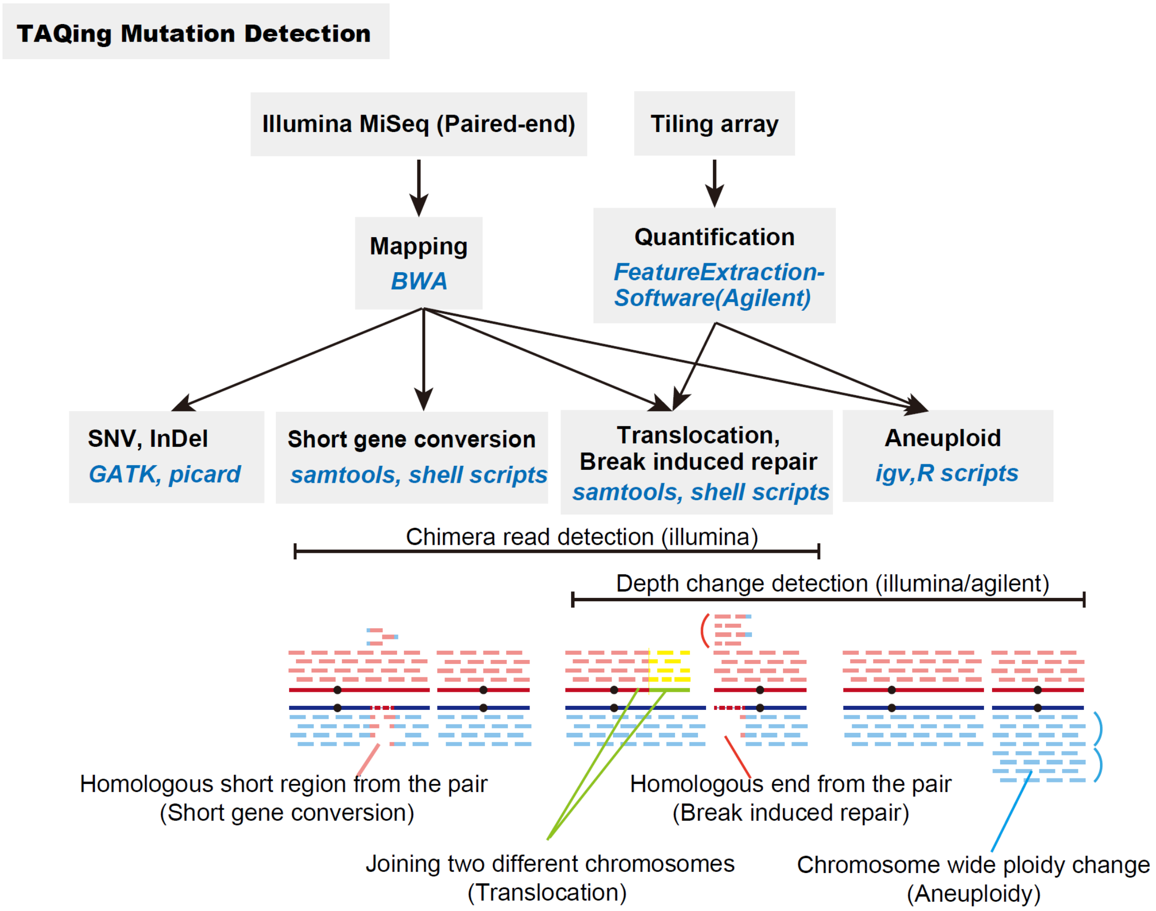

Supplementary Fig. 16. Bioinformatics workflow describing mutation detection in TAQed yeast. Genomic rearrangements were classified according to sequence features and copy number information.

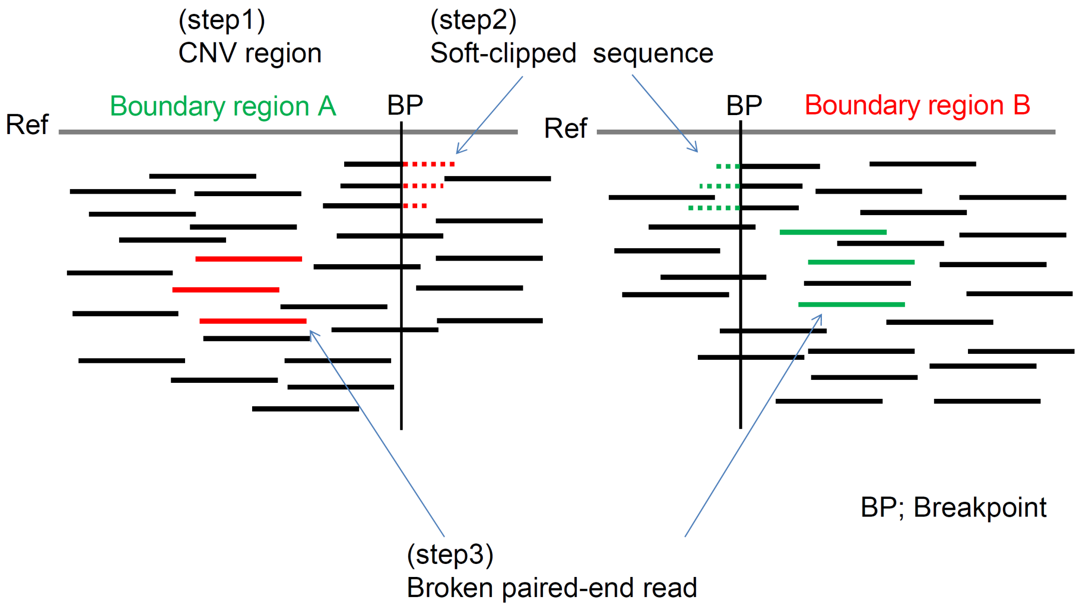

Supplementary Fig. 17. Validation of structural variants. To verify structural variants, we adopted a strategy involving three-step breakpoint confirmation, detection of CNV region using variant-call tools, and collection of soft-clipped sequence information, and detection of broken paired-end reads. Structural variants also detected in the control strains were excluded because of their potential as false positives.

Supplementary Table 1-1. Structural variants of yeast strains.

strain	rearrangement type	chromsome
81	Chromosomal duplication	YPH499Chr05
113	Chromosomal deletion	S799Chr01
113	Chromosomal deletion	YPH499Chr05
117	Chromosomal deletion	S799Chr05
119	Chromosomal deletion	YPH499Chr08

strain	rearrangement type	chromsome	position	mutation
1	insertion	YPH499Chr14	307525	G->GA
1	SNP	YPH499Chr15	470253	A->G
73	SNP	S799Chr11	286372	G->C
76	SNP	S799Chr15	773938	A->G
76	SNP	S799Chr15	979734	G->A
81	SNP	YPH499Chr11	109779	C->T
83	SNP	YPH499Chr01	153350	G->C
84	SNP	YPH499Chr02	584251	G->A
84	SNP	YPH499Chr06	195908	C->T
84	insertion	YPH499Chr07	698814	C->CA
97	SNP	YPH499Chr07	211054	C->G
97	SNP	YPH499Chr07	1083671	C->A
97	insertion	S799Chr15	96926	T->TATA
97	insertion	YPH499Chr06	187330	G->GATA
101	SNP	S799Chr08	159700	A->G
101	SNP	S799Chr09	391001	G->A
101	SNP	YPH499Chr05	495128	G->A
101	SNP	YPH499Chr05	495149	C->T
101	insertion	S799Chr10	336446	G->GA
101	insertion	S799Chr12	636809	C->CA
107	SNP	YPH499Chr04	138021	C->A
107	SNP	YPH499Chr04	138034	A->C
117	SNP	S799Chr13	208484	G->T

strain	rearrangement type	chromsome	start	end
1	SGC	YPH499Chr11	352300	358300
73	SGC	S799Chr02	673200	681200
73	SGC	S799Chr04	180000	188500
73	SGC	S799Chr04	1320800	1323800
73	SGC	YPH499Chr04	180600	181600
83	deletion	S799Chr12	857796	857822
84	SGC	S799Chr04	1207300	1215000
84	SGC	YPH499Chr06	204000	209500
84	SGC	YPH499Chr07	694300	698600
84	SGC	S799Chr11	563400	577100
84	SGC	YPH499Chr13	454000	480000
84	SGC	YPH499Chr14	501600	516700
84	SGC	S799Chr15	58800	73400
84	SGC	YPH499Chr16	693000	697000
97	SGC	YPH499Chr06	154200	156400
101	SGC	YPH499Chr02	324500	327800
101	SGC	S799Chr05	485600	488700

Blue data represent the approximate rearrangement regions, whereas black data represent precise positions. (SNP; Single Nucleotide Polymorphisms, SGC; Short Gene Conversion, BIR; Break induced repair, TL; translocation)

Supplementary Table 1-2. Structural variants of yeast strains.

strain	rearrangement type	chromsome	start	end
101	SGC	S799Chr09	379600	390600
101	SGC	YPH499Chr11	355400	385400
101	SGC	YPH499Chr14	316400	320000
106	SGC	S799Chr15	366300	369900
106	SGC	S799Chr15	775000	780000
107	SGC	S799Chr02	490400	496800
107	SGC	YPH499Chr04	134300	137700
107	SGC	S799Chr15	959600	972700
107	SGC	YPH499Chr14	596600	620900
107	SGC	YPH499Chr16	162500	175200
107	SGC	YPH499Chr16	885300	888600
113	SGC	S799Chr06	212000	219500
113	SGC	YPH499Chr11	46900	54500
113	SGC	YPH499Chr11	347000	348000
113	SGC	YPH499Chr11	633000	635200
113	SGC	S799Chr11	277600	278400
113	SGC	S799Chr07	159300	160000
113	SGC	YPH499Chr10	419800	421700
113	SGC	S799Chr13	373800	378000
113	SGC	S799Chr15	19800	22000
113	SGC	S799Chr15	44300	46300
113	SGC	S799Chr15	909000	912600
119	SGC	YPH499Chr11	330800	333900
119	SGC	YPH499Chr10	167000	168000

strain	rearrangement type	Left arm	Right arm	rearranged position
1	BIR	S799Chr04	YPH499Chr04	987500
1	BIR	S799Chr14	YPH499Chr14	280500
83	BIR	YPH499Chr02	S799Chr02	264600
83	BIR	YPH499Chr12	S799Chr12	rDNA
84	BIR	S799Chr16	YPH499Chr16	700100
97	BIR	YPH499Chr11	S799Chr11	316500
101	BIR	S799Chr07	YPH499Chr07	929500
101	BIR	YPH499Chr09	S799Chr09	385400
106	BIR	S799Chr12	YPH499Chr12	rDNA
106	BIR	YPH499Chr14	S799Chr14	649000
106	BIR	YPH499Chr14	S799Chr14	649000
107	BIR	S799Chr15	YPH499Chr15	114500

strain	rearrangement type	chromsome	region	chromsome	region
73	TL (homologous)	S799Chr12	rDNA	YPH499Chr12	rDNA
76	TL (nonhomologous)	S799Chr06	134761	YPH499Chr06	33331
76	TL (nonhomologous)	S799Chr12	78284	S799Chr08	210916
81	TL (nonhomologous)	S799Chr05	361265	YPH499Chr15	420401
84	TL (homologous)	YPH499Chr04	1000000	S799Chr16	773500
101	TL (nonhomologous)	YPH499Chr04	445948	YPH499Chr13	260552
106	TL (nonhomologous)	YPH499Chr14	674104	YPH499Chr04	295155
107	TL (nonhomologous)	YPH499Chr15	47643	S799Chr14	622638
117	TL (homologous)	S799Chr10	410800	S799Chr15	945200

Blue data represent the approximate rearrangement regions, whereas black data represent precise positions. (SNP; Single Nucleotide Polymorphisms, SGC; Short Gene Conversion, BIR; Break induced repair, TL; translocation)

Supplementary Table 2. Structural variants of TQ4 plants.

sample	SV no.	rearrangement type	region	TaqI	Transposons on breakpoint	Transposon super family
TQ4_b	SV1	Inter-chromosomal	Chr1:23227210	0	ND	
			Chr5:16328954	0	AT5TE58950	RC/Helitron
	SV2	Deletion	Chr3:38491	0	ND	
			Chr3:524640	0	ND	
	SV4	Inter-chromosomal	Chr3:524640	0	ND	
			Chr4:1476226	0	ND	
TQ4_c	SV1	Deletion	Chr1:3047208	0	ND	
			Chr1:5077993	0	ND	
TQ4_e	SV1	Inter-chromosomal	Chr1:7401166	0	ND	
			Chr4:17530659	0	ND	
	SV2	Deletion	Chr2:4022542	169	AT2TE17040	RC/Helitron
			Chr2:4291943	32	AT2TE18015	LTR/Gypsy
	SV4	Intra-chromosomal	Chr4:2020501	0	ND	
			Chr4:17530659	0	ND	
TQ4_d	SV1	Inter-chromosomal	Chr1:101475	315	ND	
			Chr2:13702700	18	ND	
	SV2	Inter-chromosomal	Chr1:101880	720	ND	
			Chr2:11519791	33	ND	
	SV3	Inter-chromosomal	Chr1:105950	2	ND	
			Chr2:10649312	10	AT2TE45655	DNA/HAT
	SV4	Inter-chromosomal	Chr1:2667845	573	ND	
			Chr2:13319792	37	ND	
	SV5	Inter-chromosomal	Chr3:627333	0	ND	
			Chr4:17055078	0	ND	
	SV7	Duplication (tandem)	Chr5:508246	0	ND	
			Chr5:2845500	0	ND	

SV: structural variant; ND: not detected.

Supplementary Data 1. Variant and strain lists of TQ2 and TQ4 plants.
->Excel file

Supplementary Data 2. Strain lists of yeasts and plants used in this study.
->Excel file

Supplementary Data 3. Statistics associated with yeast haploid-genome assembly and mapping information.
->Excel file
