# Supplementary material for: Phenotypic diversification by enhanced genome restructuring after induction of multiple DNA double-strand breaks
Source: Nat Commun. 2018 May 18;9:1995. doi: 10.1038/s41467-018-04256-y (PMC5959919; doi:10.1038/s41467-018-04256-y)
Supplement: Supplementary file 3 — Description of Additional Supplementary Files [file 41467_2018_4256_MOESM3_ESM.pdf]

Description of Additional Supplementary Files

File Name: Supplementary Data 1
Description: Variant and strain lists of TQ2 and TQ4 plants.

File Name: Supplementary Data 2
Description: Strain lists of yeasts and plants used in this study.

File Name: Supplementary Data 3
Description: Statistics associated with yeast haploid-genome assembly and mapping information.
